# Regulation of p27 (Kip1) by Ubiquitin E3 Ligase RNF6

**DOI:** 10.3390/pharmaceutics14040802

**Published:** 2022-04-06

**Authors:** Dhanraj Deshmukh, Jin Xu, Xi Yang, Hermela Shimelis, Shengyun Fang, Yun Qiu

**Affiliations:** 1 Department of Pharmacology, University of Maryland School of Medicine, Baltimore, MD 21201, USA; dhanrajdeshmukh@umaryland.edu (D.D.); jinxu@umaryland.edu (J.X.); xyang@som.umaryland.edu (X.Y.); hshimelis@gmail.com (H.S.); 2 Center for Biomedical Engineering and Technology, Department of Physiology, University of Maryland School of Medicine, Baltimore, MD 21201, USA; sfang@som.umaryland.edu; 3 Veterans Affairs Medical Center, 10 North Greene Street, Suite 3D-150, Baltimore, MD 21201, USA

**Keywords:** RNF6, p27, cell cycle, prostate cancer

## Abstract

The cyclin-dependent kinase inhibitor p27 (Kip1) is an important regulator of the G1/S checkpoint. It is degraded by the SCF-SKP2 complex in late G1 thereby allowing cells to progress to the S phase. Here we investigated the role of the E3 ubiquitin ligase RNF6 (Ring Finger Protein 6) in cell cycle progression in prostate cancer cells. Our data demonstrate that RNF6 can promote cell cycle progression by reducing the levels of p27. Knockdown of RNF6 led to an increase in the stability of p27 and to the arrest of cells in the G1 phase. RNF6 interacted with p27 via its KIL domain and this interaction was found to be phosphorylation independent. RNF6 enhanced ubiquitination and subsequent degradation of p27 in the early G0/G1 phase of the cell cycle. Knockdown of RNF6 expression by short hairpin RNA led to inhibition of the CDK2/Cyclin E complex thereby reducing phosphorylation of Retinoblastoma protein (Rb) and to a subsequent decrease in cell cycle progression and proliferation. Our data suggest that RNF6 acts as a negative regulator for p27^kip1^ leading to its proteasome-dependent degradation in the early G0/G1 phase of the cell cycle.

## 1. Introduction

RNF6 (Ring Finger Protein 6) belongs to the RING (Really Interesting New Gene) domain-containing ligase family and shares considerable homology with RNF12 (Ring Finger Protein 12) [1]. It is a 685 amino acid protein encoded by the *RNF6* gene located on chromosome 3q12 [2]. RNF6 has been reported to ubiquitinate and degrade LIMK1 (LIM Domain Kinase 1) in neuronal cells [3]. LIMK1 is a serine-threonine kinase that phosphorylates actin-binding/depolymerizing factors cofilin 1 and 2 and destrin thereby stabilizing the actin cytoskeleton. By degrading LIMK1, RNF6 is involved in regulating the local growth cone dynamics in axonal growth cones. Previous work in our laboratory has shown that RNF6 is significantly up-regulated in a subset of hormone-resistant prostate cancer patients [4]. We also demonstrated that RNF6 ubiquitinates AR and increases AR transcription under androgen-deprived conditions. Recently, it was found that RNF6 ubiquitinates p53 thereby decreasing the levels of p53 [5]. In mouse Sertoli cells, it was found that RNF6 binds to the repeat(s) of the GGGGC motif found in the promoter of the inha gene that encodes the alpha subunit of inhibin [6]. These studies demonstrate that RNF6 is involved in a number of physiological processes including axonal growth, AR transcription, transcription of inhibin, and cell cycle checkpoint modulation depending on the growth conditions and the cell type involved.

P27 also known as KIP1 is encoded by the *CDKN1B* gene. It has the cyclin and CDK (Cyclin Dependent Kinase) binding sites in the N-terminal and the QT (Amino Acids Q and T-containing) domain in the C-terminus. The former domains are involved in binding to the cyclins/CDKs whereas the latter QT domain is involved in protein stability [7]. It is involved in tumor suppression and regulates G1 to S phase transition during the cell cycle. It binds to and regulates the activity of cyclin-dependent kinases (Cdks) 1 and 2. In G0 and early G1, p27 translation and protein stability are maximal and it binds and inhibits the cyclin E–CDK2 complex [8,9,10]. The progressive decrease in p27 during G1 allows cyclin E–CDK2 and cyclin A–CDK2 to activate the transcription of genes that are required for the G1–S transition and to participate in the initiation of DNA replication [11]. D-type cyclin–Cdks are activated by mitogens to cause G0 exit and G1 progression. p27 has a dual role in the regulation of cyclin D–CDK4 and cyclin D–CDK6 complexes. In early G1 to mid-G1, p27 also promotes the assembly and nuclear import of cyclin D1–Cdk complexes. Tyrosine phosphorylation of p27 leads to the activation of assembled cyclin D–Cdks [12,13]. Knockout of p27 in mice leads to multi-organ hyperplasia, increased body size, and increases susceptibility to carcinogen-induced tumors which suggests that p27 acts as a tumor suppressor and controls both tissue growth as well as cell proliferation [14,15]. Lower p27 levels correlate with poor prognosis in a number of cancers. In prostate cancer, seven out of nine analyses involving 1464 patients showed that reduced nuclear p27 is an independent predictor of decreased time between prostatectomy and biochemical recurrence [16]. p27 is rarely mutated or deleted in human cancers [17]; however, p27 levels are reduced or the protein is mis-localized to the cytoplasm. The best-known proteolytic mechanism of p27 is by the E3 ligase complex SCF^SKP2^ which ubiquitinates and degrades p27 when it is phosphorylated on Thr187 [18]. SKP2 might also be involved in phosphorylation independent proteolysis of p27 in G1 [19,20]. However, it is important to note that expression of Skp2 begins in the S phase, except for Skp2-overexpressing tumors [21,22]. Moreover, Skp2-independent degradation of p27 was observed in Skp2-knockout lymphocytes [23]. Therefore, ubiquitin-mediated degradation of p27 might be mediated by more than one E3 ligase. We previously showed that knockdown of RNF6 expression led to the inhibition of cell proliferation through regulating AR activity [4]. However, in AR-negative PC3 cells, knockdown of RNF6 also led to growth inhibition concomitant with a decreased level of p27, suggesting a role of RNF6 in cell cycle regulation in an AR-independent manner.

In this report, we demonstrate that RNF6 can promote cell cycle progression by interacting with p27 via its KIL domain and enhance p27 ubiquitination and degradation in the early G0/G1 phase of the cell cycle. in prostate cancer cells. Our findings suggest a novel role of RNF6 in cell cycle regulation and provide new insights into mechanisms underlying prostate cancer progression.

## 2. Materials and Methods

### 2.1. Cell Lines and Cell Culture

Cell lines were purchased from American Tissue Culture Collections (ATCC, Manassas, VA, USA). CWR-R1 cells were a kind gift from Drs. Gregory and Wilson of the University of North Carolina Chapel Hill [24]. Cells were maintained in a 37 °C incubator at 5% CO_2_. 293T cells were cultured in DMEM (Cellgro® Mediatech, Manassas, VA, USA) supplemented with 10% fetal bovine serum (FBS) and 1% streptomycin/penicillin. LNCaP, CWR-R1 and PC3 cells were cultured in RPMI 1640 medium (Cellgro® Mediatech, Manassas, VA, USA) with 10% FBS and 1% penicillin/streptomycin. Cell proliferation was assayed using CCK-8 (Dojindo Molecular Technologies Inc., Rockville, MD, USA).

### 2.2. Constructs

RNF6 constructs were cloned as described previously [4]. The RNF6 KIL domain mutant and p27T187A phosphomutant were made using the QuickChange Mutagenesis Kit (Stratagene, San Diego, CA, USA). The primers used for mutagenesis were listed in Table 1. All mutations were confirmed by sequencing. *RNF6* shRNAs were constructed as described previously [25]. The target sequences of the human *RNF6* shRNAs (shorthairpin RNAs) are 5′-CCTCAGTGAATTTCAATGGTA-3′ (shRNF6-1) and 5′-CCATAACAGTTCCTCTTCGTA-3′ (shRNF6-2). The GST-p27 plasmid was a kind gift from Dr. Arnaud Besson (CBI, Toulouse, France).

### 2.3. In Vitro Ubiquitination

In vitro ubiquitination assay was carried out as described previously with minor modifications [26,27]. Briefly, GST-tagged RNF6 (aa 247–685) or the mutant GST-RNF6 (aa 247–685—frameshift mutation in the RING domain) was expressed in *E. coli* cells and then affinity-purified with glutathione-sepharose 4B beads. Ten nanograms of purified GST-p27 was mixed with 100 ng of either ligase-active or the ligase-inactive GST-RNF6 in a total volume of 25 μL containing 50 mM HEPES (pH7.9), 4 mM ATP, 5 mM MgCl_2_, 15 μM ZnCl_2_, (Sigma-Aldrich, Saint Louis, MO, USA) 150 μM ubiquitin (Boston Biochem, Cambridge, UK, Cat. #U-100H), 30 nM rabbit E1 (Boston Biochem, Cambridge, UK, Cat. #E302), and 200 nM UbcH10 (Boston Biochem, Cambridge, UK, Cat. #E2-650). The reaction was incubated at 37 °C for 4 h and reactions were terminated by the addition of SDS sample buffer. Samples were resolved by SDS-PAGE and immunoblotted with anti-p27 antibody. In some experiments, the E2 UbcH10 was replaced with UbcH2, UbcH5a, or Use1 (Boston Biochem, Cambridge, UK).

### 2.4. Cell Cycle Analysis

Cell cycle analysis was carried out using the protocol by Riccardi et al. [28], with slight modification. Harvested cells were fixed in 70% cold ethanol and then stored at 20 °C at least overnight. Fixed cells were washed with PBS and then resuspended in 0.25 mL of PBS. Five microliters of 10 mg/mL of RNase A was added to the suspension and the cells were incubated for 1 h at room temperature. Ten microliters of 1 mg/mL propidium iodide solution was then added and the cells were stored in dark at 4 °C until analysis.

### 2.5. Transient Transfections

Cells were plated in 6-well plates or 100-mm dishes for 24 h before transfection. 293T cells were transfected using a calcium phosphate transfection kit (mammalian transfection kit, CalPhos™, Takara Bio Europe, Saint-Germain-en-Laye, France). Lysates of cells were collected ∼48 h post-transfection.

### 2.6. Western Blotting, Immunoprecipitation and Antibodies

For Western blots, cell lysates were prepared in RIPA (Radioimmunoprecipitation assay) buffer supplemented with protease inhibitors and the protein concentrations were quantified using the Bradford method [29]. Proteins were resolved on 8–15% SDS-polyacrylamide gels by electrophoresis, transferred to a PVDF (Millipore, Burlington, MA, USA) membrane, non-specific binding blocked and the protein probed with an antibody and then detected by chemiluminescence. For immunoprecipitations (IPs), cell lysates were collected using IP buffer as described previously [30]. IPs were carried out by adding ∼1–2 μg of antibody per mL cell lysates incubated overnight at 4 °C and the antibody conjugated to the protein was pulled down by using recombinant protein A Sepharose conjugated beads. The beads were washed and the proteins were eluted in 50–100 µL SDS loading buffer. For detecting ubiquitinated proteins, lysates were prepared under denaturing conditions prior to IP [31]. Antibodies used for Western blotting, immunoprecipitation, and immunofluorescence included p27 (sc53871 and sc528), Ub(Ubiquitin)(sc8017), GAPDH (sc47724), actin (sc7210), p-p27 (Thr187) (sc16324-R), p-p27 (Ser10) (sc12939), CDK2 (SC163), Cyclin D1 (sc-8396), PARP-1 (sc-8007), cyclin B1 (sc245) from Santa Cruz (Santa Cruz, CA, USA) and cyclin E1 (#4129), SKP2 (#4358), Rb (#9309), p-Rb (#8516) from Cell Signaling (Danvers, MA, USA). The HA antibody was from Covance (Madison, WI, USA) (MMS-101P). The monoclonal antibody for RNF6 (anti-RNF6) was developed by immunizing mice with a purified fusion protein containing N-terminal residues of RNF6 (aa 1–246), and hybridoma clones were isolated and maintained in RPMI 1640 medium containing 25 mM of HEPES (Invitrogen, Waltham, MA, USA). The conditioned medium was used in the study.

### 2.7. Quantitative Real-Time RT-PCR

RNA was extracted using TRIzol reagent (Invitrogen, Waltham, MA, USA). Total RNA was treated with DNase (Promega RQ1 kit, Madison, WI, USA) and transcribed into cDNA (Roche Transcriptor Reverse Transcriptase kit, Sigma-Aldrich, Saint Louis, MO, USA), both according to manufacturers’ protocols. PCR was carried out using the Roche FastStart High Fidelity PCR Kit (Sigma-Aldrich, Saint Louis, MO, USA). Real-time PCR was carried out using the Roche FastStart SYBR Green Master Kit (Sigma-Aldrich, Saint Louis, MO, USA). 18S rRNA primers were TTGACGGAAGGGCACCACCAG (forward) and GCACCACCACCCACGGAATCG (reverse); p27 primers were ATCACAAACCCCTAGAGGGCA (forward) and GGGTCTGTAGTAGAACTCGGG (reverse). The relative abundance of each transcript was quantified by using the ΔΔCt formula using 18S rRNA as an internal control.

### 2.8. In Vitro Cell Proliferation Assays

Cells were plated in 100 mm plates at a density of ∼1 × 10^6^ cells/plate 24 h prior to lentiviral infection. As vector-treated cells reached confluence, cells were fixed with 1% formaldehyde (Sigma-Aldrich, Saint Louis, MO, USA) for 1 h, and then stained with Coomassie Blue dye for 1 h.

### 2.9. Immunofluorscence Staining

CWR-R1 cells were plated in Millipore chamber slides (Millicell^®^ EZ, Sigma-Aldrich, Saint Louis, MO, USA) and transduced with either control shRNA or shRNF6-1/2. After about 72 h, cells were washed with PBS and then fixed in 4% formaldehyde for 15 min. Cells were washed 3× with 1× PBS and then blocked in blocking solution (1× PBS/1% BSA/5% Normal Donkey Serum/0.3% Triton^TM^ X-100, Sigma-Aldrich, Saint Louis, MO, USA) for 1 h. Cells were immunostained with primary antibody (RNF6 mouse monoclonal Ab 1:100; p27 rabbit Ab 1:200) in blocking buffer for 2 h at room temperature. Cells were washed 3× with 1× PBS and then incubated with Alexa 488-conjugated anti-rabbit antibody (1:400) and Cy5-conjugated anti-mouse antibody (1:300) (Invitrogen, Waltham, MA, USA) for 1 h at room temperature. Slides were washed, counterstained with DAPI (4′,6-diamidino-2-phenylindole), coverslip mounted with anti-fade Vectashield (Vector Laboratories, Burlingame, CA, USA), and then observed under a confocal microscope.

### 2.10. Cytoplasmic Nuclear Fractionation

The cytoplasmic nuclear fractions were prepared as described by Holden et al. [32] with slight modifications. Briefly, cells were transduced with either control shRNA or shRNF6-1/2. After 72 h, cells were trypsinized and washed with 1× PBS and then incubated with an appropriate volume of cytosolic buffer (150 mM NaCl, 50 mM HEPES pH 7.4, 25 µg/mL digitonin, protease inhibitors) for 10 min at 4 °C with gentle agitation. The suspension was pelleted and the supernatant was collected as the cytosolic fraction. The pellet was washed once with the cytosolic buffer and then lysed in RIPA buffer containing protease inhibitors. The lysates were pelleted at g for 15 min and the supernatant was collected as the nuclear fraction. Protein concentration was estimated in each sample and then a 5× SDS loading buffer was added. Lysates were denatured by boiling at 95 °C for 5 min. and then subjected to Western blot analysis.

### 2.11. Statistical Analyses

Experiments were performed a minimum of three replicates. Statistical analyses were performed as a Student’s *t*-test or ANOVA followed by a post hoc Dunnett’s multiple comparison test using GraphPad Prism software (Version 5) (GraphPad Software, Inc., San Diego, CA, USA). *p*-values of *p* < 0.05 were considered statistically significant.

## 3. Results

### 3.1. Knockdown of RNF6 Leads to Increased Stability of p27

**Figure 1:**
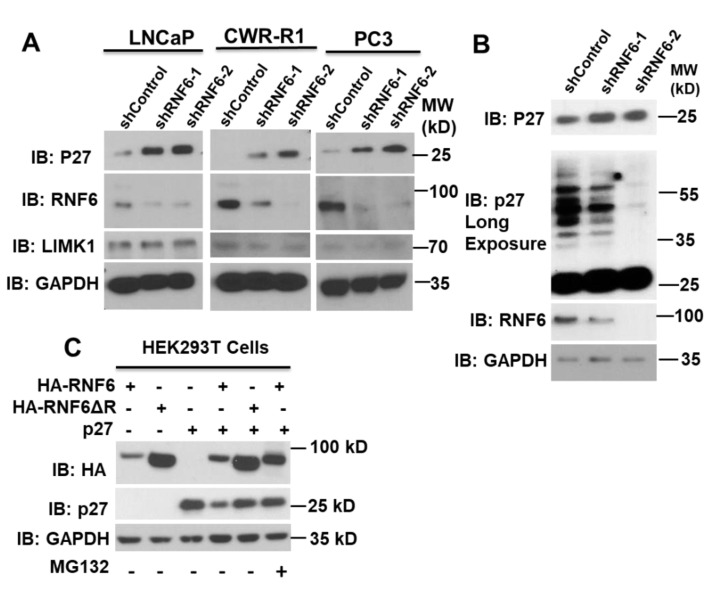
Knockdown of RNF6 leads to increase in stability of p27. (**A**) Prostate cancer cell lines were transduced with either control shRNA or shRNF6-1/2. After 72 h of knockdown, cells were lysed and lysates subjected to western blot analyses. (**B**) LNCaP cells were transduced with either control shRNA or shRNF6 1/2. After 72 h, cells were lysed and lysates subjected to Western blot analysis. (**C**) HEK293 cells were transfected with the indicated plasmids by the CaCl2 method. After 42 h, MG132 (10 µM) was added as shown. After 48 h of transfection, cells were lysed in RIPA buffer with protease inhibitors. The lysates were then subjected to western blot analysis.

To examine the effects of RNF6 knockdown on cell cycle regulator proteins, three prostate cancer cell lines were used viz; LNCaP (AR dependent), CWR-R1 (androgen independent), and PC3 (AR negative). Knocking down of RNF6 by shRNA in CWR-R1, LNCaP, and PC3 cells led to an accumulation of p27 protein (Figure 1A), while the levels of LIMK1, a previously known RNF6 substrate in neuronal cells [3], were unaffected by RNF6 knockdown in these cells (Figure 1A), suggesting a cell context-dependent regulation. The increased p27 protein level in RNF6 knockdown cells was inversely proportional to the higher molecular weight ubiquitinated p27 shown by a longer exposure of the blot (Figure 1B). Since RNF6 is a ubiquitin E3 ligase, we hypothesized that p27 could be a potential substrate of RNF6. To test this hypothesis, we transfected HEK293T cells with either p27 along with HA-tagged wild-type WT-RNF6 (aa 1-685) or with the ΔRING mutant (aa 1–629) (Figure 1C). When p27 was co-expressed with WT-RNF6, there was a significant decrease in p27 levels, while the mutant RNF6 (ΔRING) had little effect on p27 expression. When cells were treated with the proteasome inhibitor, MG132 (10 µM), the p27 protein level resumed indicating that the decrease in p27 levels was due to proteasomal degradation. These data suggest that p27 could be a potential target for ubiquitin-mediated proteasomal degradation by RNF6.

### 3.2. RNF6 Interacts with P27 via Its KIL Motif in a Phosphorylation-Independent Manner

**Figure 2:**
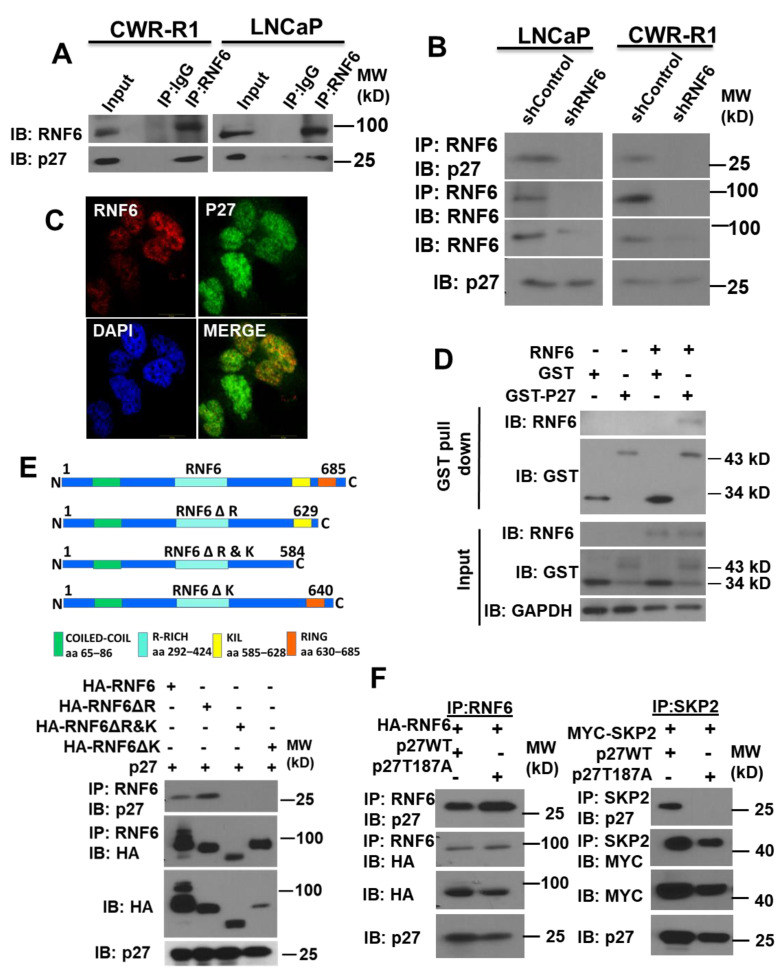
RNF6 interacts with p27 in a phosphorylation independent manner. (**A**) Prostate cancer cells were treated with MG132 (10 µM) for 6 h and then lysed in IP buffer. Lysates were immunoprecipitated with anti-RNF6 antibody and immunoblotted for p27. Preimmune IgG was used as control. (**B**) LNCaP and CWR-R1 cells were transduced with either control or RNF6 shRNA. After about 66 h post-transduction, cells were treated with MG132 (10 µM) for 6 h. Cells were then lysed in IP buffer and the lysates immunoprecipitated with anti-RNF6 antibody. Immunoprecipitates were then immunoblotted for p27. RNF6 blots were used as control. (**C**) Immunofluorescence confocal microscopy was carried out in CWR-R1 cells with anti-RNF6 (Red) and anti-p27 (green) antibodies. Nuclei were counterstained with DAPI. (**D**) LNCaP cells were transfected with either GST or GST-p27 alone or with RNF6. After 48 h of transfection, cell lysates were prepared and GST immunoprecipitated with GSH beads. The immunoprecipitate was then immunoblotted for GST and RNF6. (**E**) HA-tagged WT RNF6 and various mutants were cotransfected with p27 in HEK293 cells. After 42 h, cells were treated with MG132 (10 µM) for 6 h after which they were lysed and lysates immunoblotted for HA and p27. Cartoons of WT RNF6 and different mutants. (**F**) WT RNF6 or Myc-tagged SKP2 was cotransfected with WT p27 or the phosphomutant p27T187A in HEK293 cells. Cells were treated with MG132 (10 µM) for 6 h prior to cell lysis. After 48 h of transfection, cells were lysed and lysates immunoprecipitated with anti-RNF6 antibody. Immunoprecipitates were subjected to western blot analysis and immunoblotted for HA/Myc and p27.

Since it was observed that RNF6 decreases p27 levels post-translationally, we sought to understand if these two proteins interacted with each other. The interaction between p27 and RNF6 was confirmed by co-immunoprecipitation in both LNCaP and CWR-R1 cells (Figure 2A). This interaction was lost when co-immunoprecipitation was carried out under RNF6 knockdown conditions. further confirming the specificity of the interaction (Figure 2B). Immunofluorescence staining revealed co-localization of RNF6 and p27 primarily within the nucleus (Figure 2C). The interaction between p27 and RNF6 appeared to be direct and confirmed by the GST pulldown experiments (Figure 2D). We next examined what domains were involved in the interaction between p27 and RNF6. As shown in Figure 2E, deletion of the RING domain had little effect on the interaction of two proteins, suggesting their interaction was independent of the RING domain. When additional deletion of a stretch of 45 amino acids just upstream of the RING domain, which also contains a KIL motif (a highly conserved motif in a subset of RING-H2 E3 ligases—hereby designated KIL domain), was carried out, RNF6 no longer bound to p27. Deletion of the KIL domain of RNF6 completely abolished its binding to p27. Taken together, these results revealed that the KIL domain was essential for RNF6 to bind with p27. Since the interaction between SKP2 and p27 is phosphorylation dependent, we investigated whether the interaction between RNF6 and p27 is also regulated by phosphorylation. As shown in Figure 2F, both WT p27 and its phosphorylation-deficient mutant T187A could be co-immunoprecipitated with RNF6, indicating that the interaction between RNF6 and p27 was not dependent on the phosphorylation of T187, while the p27 T187A mutant failed to co-immunoprecipitate with SKP2 as expected.

### 3.3. RNF6 Knockdown Leads to Accumulation of P27 in the Nucleus

**Figure 3:**
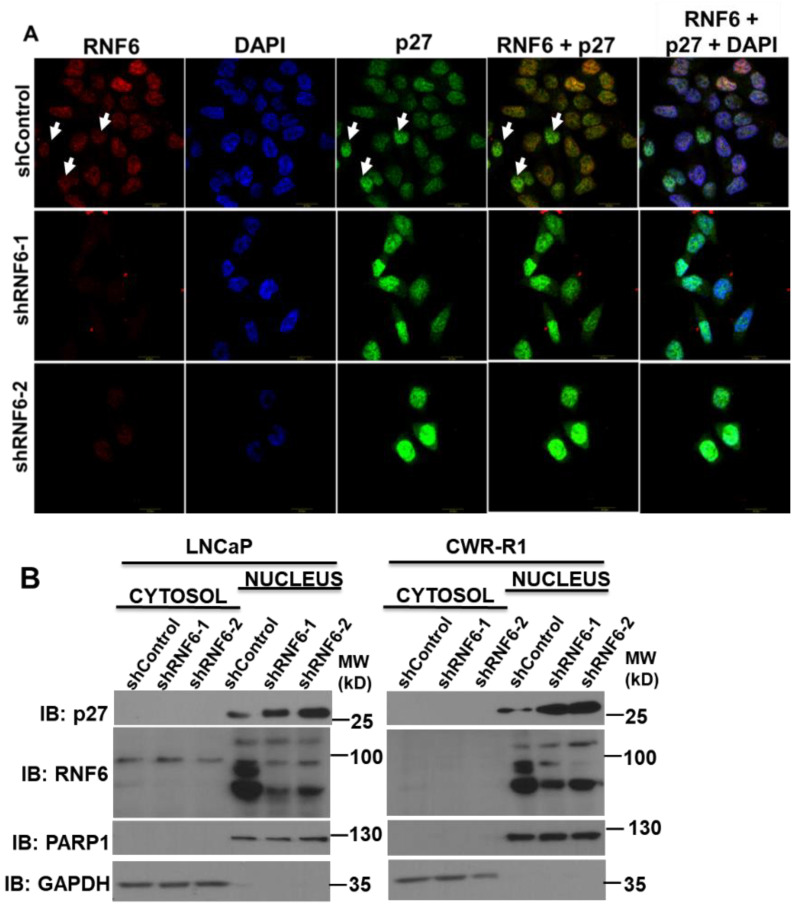
RNF6 knockdown leads to increase in p27 levels in the nucleus. (**A**) CWR-R1 cells were transduced with either control shRNA or shRNF6. After 72 h, cells were fixed, and immunofluorescence confocal microscopy was carried out on the cells by co-staining with anti-p27 (green) and anti-RNF6 (red) antibodies. Cells were counterstained with DAPI. (**B**) LNCaP/CWR-R1 cells were transduced with either control shRNA or shRNF6. After about 72 h of transduction, cells were fractionated as described in the Materials and Methods. The cytosolic and nuclear fractions were then subjected to Western blot analysis.

p27 is frequently mis-localized to the cytoplasm in a number of cancers [33,34]. Cytosolic p27 has been implicated in a number of oncogenic processes such as metastasis and resistance to cancer therapy. In order to investigate if abrogation of RNF6 led to mis-localization of p27, we prepared cytosolic and nuclear fractions of WT and RNF6 KD LNCaP and CWR-R1 cells. Since p27 is a small protein, cell fractionation using detergents such as Triton X-100 leads to its leakage into the cytosol [35]. Hence, we used a buffer containing a mild detergent such as digitonin as discussed in the Materials and Methods. Immunoblotting of the cytosolic and nuclear fractions revealed that accumulation of p27 after RNF6 knockdown was primarily confined to the nucleus (Figure 3B). Moreover, RNF6 too was mainly concentrated within the nucleus. To confirm these observations, we performed fluorescence microscopy in CWR-R1 cells infected with either control or RNF6-specific shRNA. We observed that the knockdown of RNF6 (red) led to an increase in the levels of p27 (green) and that this increase in p27 was mostly nuclear (Figure 3A). These observations corroborated with the cell fractionation experiments. Thus, abrogation of RNF6 leads to the accumulation of p27 within the nucleus.

### 3.4. RNF6 Polyubiquitinates P27 In Vivo and In Vitro

**Figure 4:**
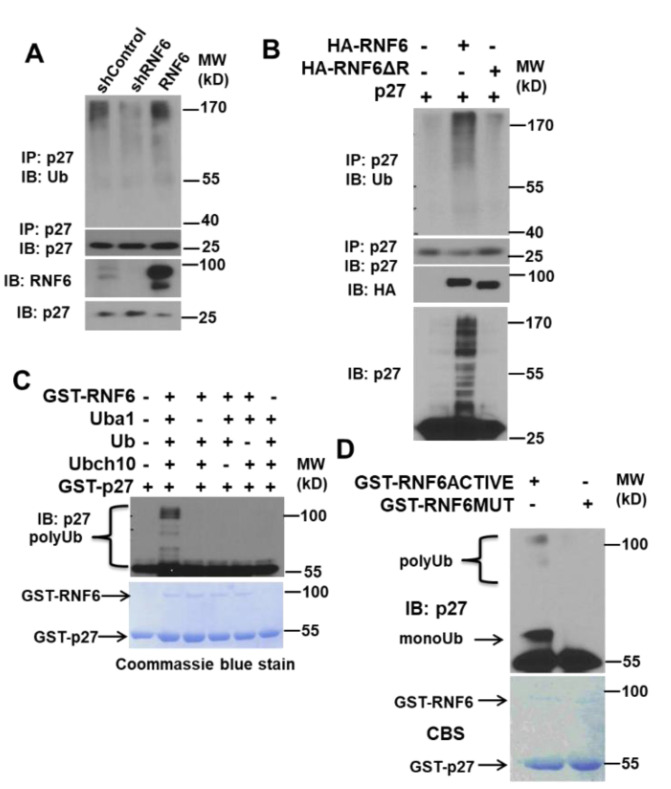
RNF6 ubiquitinates p27 in vivo and in vitro. (**A**) LNCaP cells were transduced with scrambled shRNA, shRNF6, or RNF6 plasmid. After about 72 h of transduction, cells were lysed and lysates immunoprecipitated with anti-p27 antibody. Immunoprecipitates were then probed for Ub. (**B**) HEK293 cells were transfected with either p27 alone, WT-RNF6 and p27, or mutant RNF6 (ΔRING) and p27. After 42 h of transfection, cells were treated with MG132 for an additional 6 h. Cells were then lysed in denaturing buffer and lysates immunoprecipitated with anti-p27 antibody. The immunoprecipitates were immunoblotted for Ub. (**C**) The in vitro ubiquitination assays were carried out as described in Materials and Methods. Top, immunoblot of anti-p27 to detect p27. Bottom: Coomassie blue staining (CBS) of the gel to monitor the amount of E3 ligases present in the reactions. (**D**) Same as in C except that either active or inactive GST-RNF6 was used.

A number of ubiquitin E3 ligases regulate p27, temporally and spatially. The most well studied of them all is the SCF-SKP2 complex [18]. In order to study the ubiquitination of p27 by RNF6, we utilized both in vivo and in vitro systems. We transduced LNCaP cells with control shRNA, RNF6 shRNA, or RNF6-overexpressing plasmid. After about 48 h of infection, we immunoprecipitated p27 from the cell lysates and then immunoblotted with anti-Ub antibody. We observed that knockdown of RNF6 led to a decrease in p27 ubiquitination whereas overexpression of RNF6 led to a marginal increase in ubiquitinated p27 (Figure 4A). To confirm these observations, we co-expressed either WT or RING domain-deleted RNF6 mutant along with p27 in HEK293T cells. p27 alone was used as a control. After 48 h, cell lysates were prepared under denaturing conditions and subjected to immunoblotting. Co-expression of WT-RNF6 along with p27 led to a significant increase in ubiquitinated p27 as was apparent from the increase in higher molecular weight p27 observed from immunoblotting with p27 antibody. When p27 was immunoprecipitated from these lysates and then probed for Ub, a significant increase in ubiquitination was observed in cells co-expressing WT-RNF6 as compared to the mutant RNF6 or the p27 control (Figure 4B). We then investigated whether p27 ubiquitination could be recapitulated in a cell-free system with recombinant proteins. RNF6-mediated ubiquitination was observed only when all the components of the ubiquitin system were present in the reaction mixture (Figure 4C). When WT GST-RNF6 (aa 247–685) was substituted with a mutant GST-RNF6 (aa 247–685—frameshift mutation in the RING domain), the polyubiquitination of p27 was completely lost (Figure 4D). These data suggest that RNF6 ubiquitinates p27^kip1^ in vivo as well as in vitro and that p27 is a direct substrate of RNF6.

### 3.5. RNF6 Knockdown Leads to P27 Accumulation in Early G1 and to Reduced Rb Phosphorylation

**Figure 5:**
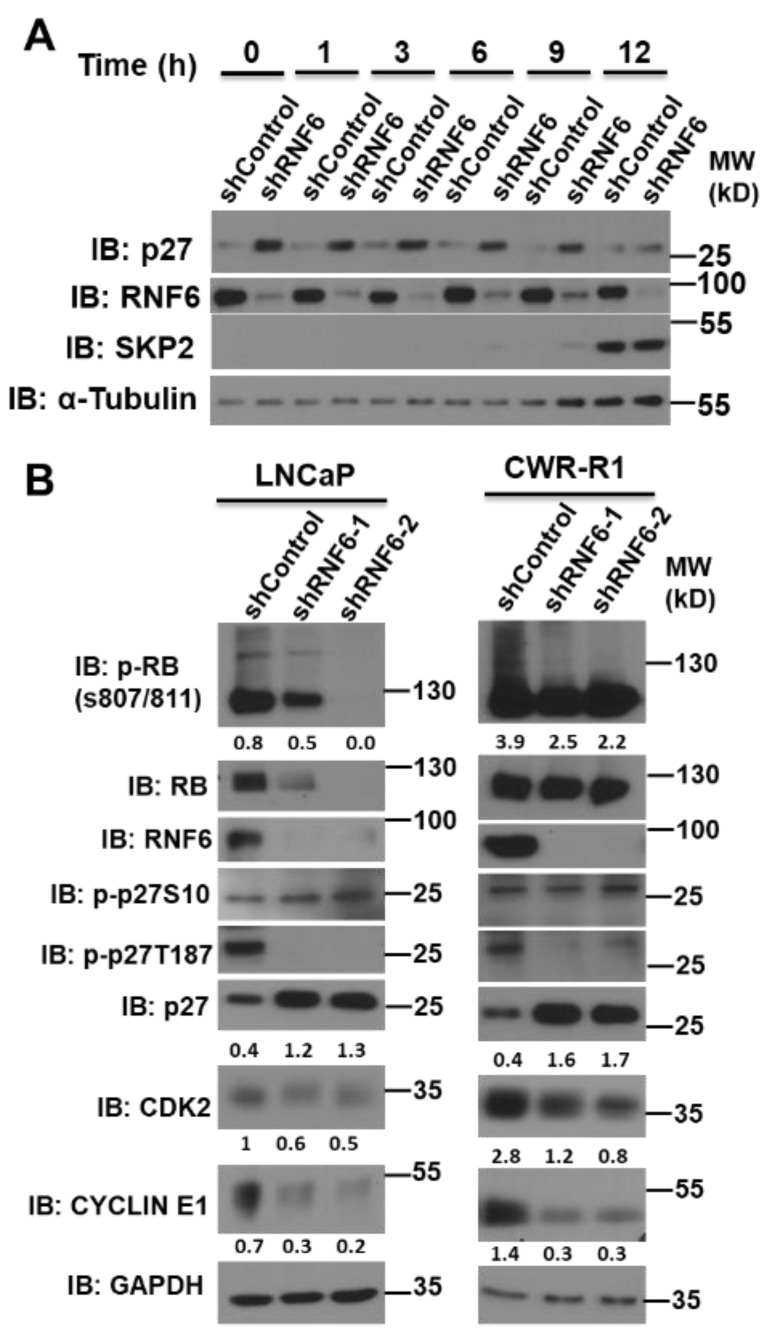
RNF6 knockdown leads to p27 accumulation in early G1 and reduced Rb phosphorylation. (**A**) CWR-R1 cells were transduced wither either control sRNA or shRNF6. After 48 h of transduction, cells were synchronized in G0/G1 by serum withdrawal for 48 h. Ten percent final serum was added back and cells were harvested at indicated time points. Cells lysates were prepared and immunoblotted as shown. (**B**) CWR-R1 and LNCaP cells were transduced with either control shRNA or shRNF6. After 72 h of transduction, cell lysates were prepared and subjected to Western blot analysis as shown.

p27 is regulated post-translationally by a number of different mechanisms. To understand the role of RNF6 in a temporal setting, we knocked down RNF6 using small hairpin RNA and studied the effect on p27 stability in G0/G1-synchronized cells. We observed that RNF6 ablation led to stabilization of p27 in the early G1 phase until 9 h. During this time, there was no expression of SKP2. At the late G1/S boundary, when SKP2 levels increased, the p27 levels reduced in both control and RNF6 knockdown cells (Figure 5A). Moreover, levels of RNF6 remained more or less constant during this period. These observations suggest that RNF6 regulates p27 levels during the early G1 phase of the cell cycle in prostate cancer cells. Since p27 levels during early G1 are governed by p27 S10 phosphorylation, we probed for changes in p27S10 phosphorylation as a result of RNF6 ablation [36]. We observed that the p27 S10 levels remain unchanged in the absence of RNF6 (Figure 5B). However, there was a decrease in cyclin E1 and CDK2 levels in both LNCaP and CWR-R1 cells. This decrease might be due to the inhibition of the cyclin E1/CDK2 complex by p27. A decrease in the cyclin E1/CDK2 levels further led to a decrease in phosphorylation of the Rb protein on S807/811 residues in LNCaP cells. Rb phosphorylation was marginal in CWR-R1 cells. The concomitant decrease in p27 T187 levels could be a direct result of the decrease in cyclin E1/CDK2 levels. These data demonstrate that RNF6 ablation leads to increased stability of p27 in the early G1 phase and consequently leads to reduced Rb phosphorylation.

### 3.6. Knockdown of RNF6 Leads to G1/S Block and to Reduced Cell Proliferation

**Figure 6:**
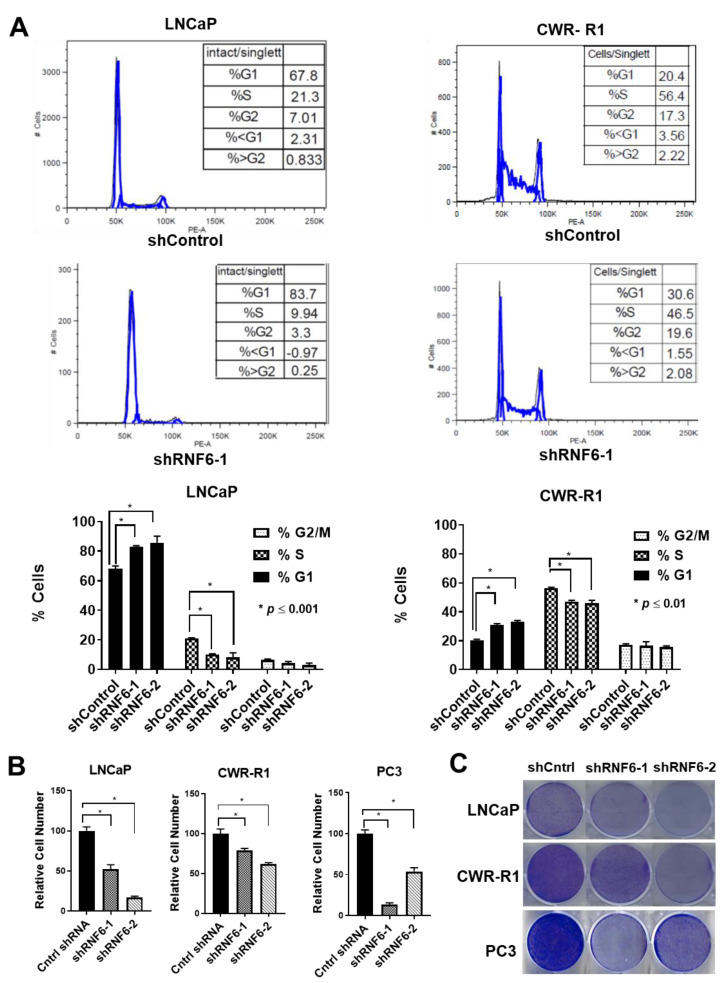
Knockdown of RNF6 leads to G1/S block and to reduced cell proliferation. (**A**) Asynchronous cells were transduced with either control shRNA or shRNF6 for 72 h after which they were stained with PI as described in materials and methods. After PI staining, cells were subjected to FACS analysis. (**B**) Cells were transduced with either control shRNA or RNF6 shRNA for 7 days. Cell proliferation was then assessed using CCK8 assay, * *p* < 0.05. (**C**) Cells were transduced with control or RNF6 shRNA as in B. Cells were then fixed and stained with Coomassie blue dye.

To understand the consequences of RNF6 depletion on cell cycle function, we performed cell cycle analysis and cell proliferation assays. When control and RNF6 knockdown asynchronously growing LNCaP and CWR-R1 cells were subjected to cell cycle analysis, it was observed that a significantly higher number of cells were stuck in G0/G1 in RNF6 knockdown cells when compared to control cells (Figure 6A). This observation was true in both the cell lines viz; LNCaP and CWR-R1. However, the effect was more pronounced in LNCaP cells. This could be because LNCaP cells inherently have a higher proportion of cells in G0/G1 (60–70%) as compared to CWR-R1 cells (20–25%). Lagging of cells in G0/G1 after RNF6 knockdown was expected as p27 blocks G1–S progression of the cells. In order to assess if the G1/S block of cells had any effect on cell proliferation, we conducted cell proliferation assays using the CCK8 kit and colony-forming assay. In both the assays, it was observed that the proliferation of cells was significantly reduced on RNF6 ablation (Figure 6B,C). Again, the cell proliferation assays corroborated with our cell cycle analysis as LNCaP cells were found to be more sensitive to RNF6 knockdown when compared to CWR-R1 cells. These data demonstrate that knockdown of RNF6 leads to cell cycle arrest in G0/G1 and this leads to reduced cell proliferation.

## 4. Discussion

Treatment for castration-resistant prostate cancer continues to elude us. Early diagnosis of CRPC could increase survival and improve quality of life [37]. In organ-confined prostate tumors, p27 expression was the only significant independent predictor of the time to biochemical recurrence after radical prostatectomy [38]. Cell cycle-dependent changes in p27 levels are largely attributed to post-translational modifications such as phosphorylation and ubiquitination ultimately leading to proteasomal degradation [7]. This regulation is tightly controlled, temporally and spatially. It has been shown that p27 undergoes ubiquitination even in Skp2-null cells [23,29]. These studies suggest that multiple E3 ligases could be involved in the regulation of p27 (Kip1) levels.

In this study, we observed a dramatic decrease in cell proliferation following knockdown of RNF6 in prostate cancer cells. This prompted us to conduct a cell cycle analysis of the cells. We found that knockdown of RNF6 led to an inhibition in cell cycle progression and that the cells were unable to progress into the S phase. To identify the exact cause of this G1/S block, we immunoblotted some of the known CDK inhibitors that block G1/S progression. This exercise revealed that the protein levels of p27 were significantly up-regulated on RNF6 knockdown (Figure 1A,B). When we probed to see if there was an interaction between these proteins, we found that RNF6 interacts with p27 (Kip1) (Figure 2). This interaction depended on a stretch of 45 aa sequence (KIL domain) which contains the KIL motif just upstream of the RING domain (Figure 2) [2]. Interestingly, when the KIL domain was deleted, the activity of RNF6 was also lost as evident by the lack of auto-ubiquitination (Figure 2E). Thus, the KIL domain is not only essential for interacting with p27 but also for the ubiquitination activity of RNF6. RNF6 ablation in multiple cell lines, including PC3 cells that are AR negative, led to an accumulation of p27 (Figure 1).

Previously, we have shown that RNF6 drives AR transcriptional activity in the absence of androgen by ubiquitination and stabilization of the AR [4]. Since we observed p27 stabilization even in PC3 cells which are AR negative, this effect is most likely independent of the function of RNF6 on AR activity. It is well documented that the ubiquitination of p27 by Skp2 is phosphorylation dependent and hence we wanted to study if this case was true for the interaction between RNF6 and p27 as well [18]. We found that the interaction between RNF6 and p27 is phosphorylation independent (Figure 2E). This could be because RNF6 regulates p27 levels early on in the G0/G1 phase when the activity of the CDK complexes is minimal.

There are a number of studies documenting the mis-localization of p27 to the cytosol [39,40,41]. Loss of nuclear p27 is seen in most cancers and is considered a marker of poor prognosis. Immunohistochemistry studies of nuclear p27 are associated with adverse outcomes in prostate cancer [14], gliomas [42], and astrocytomas [43]. Mislocalization of p27 from the nucleus to the cytoplasm has been attributed to the phosphorylation of p27 on T198 by AKT and of T157 by AKT and SGK1 [40,44,45]. Phosphorylation of p27 on T198 and T157 leads to sequestration of p27 in the cytoplasm. Some studies have shown that mislocalization of p27 to the cytoplasm was associated with increased cell migration [46,47]. One of the important events in the early G0/G1 phase of the cell cycle is the phosphorylation of p27 on Ser 10 by the Mirk/dyrk1b kinase leading to the stabilization of p27 [36]. This phosphorylation is also required for the export of p27 to the cytoplasm mediated by the chromosome maintenance 1 (CRM1)/RAs-related Nuclear protein Guanosine TriPhosphate (RanGTP) complex [48]. Once exported to the cytoplasm, p27S10 is then ubiquitinated by the KPC1/KPC2 RING E3 complex in the early G0/G1 phase [49]. Depletion of RNF6 did not have any effect on the cytoplasmic localization of p27. This could be attributed at least in part to the unchanged p27S10 phosphorylation levels after RNF6 depletion. Immunofluorescence staining of CWR-R1 cells revealed that shRNA-mediated ablation of RNF6 leads to an increase in nuclear p27 staining (Figure 3B). This observation was then confirmed by the cytosolic nuclear fractions in which p27 was detected within the nucleus (Figure 3A). The cytosolic p27 was undetectable. This could be because of very low levels in the cytoplasm. These data demonstrate that RNF6, being a predominantly nuclear protein, targets p27 within the nucleus for degradation. We next studied the ubiquitination of p27 in vivo and in vitro. We observed that RNF6 ubiquitinated p27 and that the RING domain was essential for this ubiquitination (Figure 4A). These observations were further corroborated by in vitro ubiquitination experiments (Figure 4C,D). The ubiquitin E2-conjugating enzyme UBCH10 enhanced the polyubiquitination of p27 (data not shown). To study how RNF6 regulates p27 levels temporally, we synchronized control and shRNF6-infected CWR-R1 cells by serum starvation to G0/G1. Serum was added back and cells were collected at different time points for Western blot analysis. The data revealed that RNF6 levels were fairly constant throughout the cell cycle. However, p27 levels were up-regulated in the RNF6 knockdown cells from 0 h to 9 h. During this time, Skp2 levels were not detectable. At around 12 h, robust Skp2 protein levels could be seen and the p27 levels were comparable in both control and shRNF6 KD cells (Figure 5A). These data suggest that RNF6 regulates p27 levels in the early G0/G1 phase of the cell cycle and at the G1/S boundary of the cell cycle, Skp2 levels determine the fate of p27. A similar observation was reported by Hara et al., 2001, where mitogenic stimulation of lymphocytes led to the expression of SKP2 levels 18–24 h post-stimulation coinciding with G1/S transition [39]. This is in line with most of the reported literature [49,50].

As expected, stabilization of p27 leads to reduced Rb phosphorylation (Figure 5B) [51]. Moreover, p27S10 levels remain unchanged on RNF6 knockdown suggesting that nuclear retention of p27 is not a consequence of the decrease in p27S10 phosphorylation. Higher p27 levels in RNF6-ablated samples likely inhibit the cyclin E/CDK2 complex and lead to their decrease (Figure 5B). Reduction in cyclin E/CDK2 leads to a reduction in p27T187 phosphorylation levels. These data suggest that an increase in p27 levels in early G0/G1 leads to a cascading effect on the cyclin E/CDK2 complex further reducing p27T187 phosphorylation and enhancing p27 stability [23]. A consequence of increased p27 stability is that most of the cells are arrested in the G0/G1 phase of the cell cycle as evidenced by the cell cycle analyses (Figure 6A). This is expected since p27 is a known inhibitor of the G1/S progression of cells [8]. Increased p27 stability leads to reduced cell proliferation as shown by the CCK8 cell proliferation assay and the colony-forming assays (Figure 6B,C). The effect of RNF6 ablation is seen to be more pronounced in LNCaP cells as compared to CWR-R1 cells. This could be because of the inherently lower levels of p27 in CWR-R1 cells compared to LNCaP. Moreover, in LNCaP cells, more cells in the control group are in the G0/G1 phase (45–70%) compared to CWR-R1 (20–25%). Overall, these data demonstrate that RNF6 is a novel regulator of p27 and targets it for degradation in the early G0/G1 phase of the cell cycle.

Given the importance of p27 as a prognostic marker, RNF6 expression could serve as a differential diagnostic marker between hormone-naïve and hormone-resistant prostate cancers.

## Figures and Tables

**Table 1:** List of primers used for mutagenesis PCR.

Mutant	Sequences
P27T187A	GTTCTGTGGAGCAGGCGCCCAAGAAGCCTG sense
	CAGGCTTCTTGGGCGCCTGCTCCACAGAAC antisense
RNF6ΔKIL	TGAAACTGGAACACTACCCAAAATCTGTAGTGTTTGTA sense
	TACAAACACTACAGATTTTGGGTAGTGTTCCAGTTTCA antisense

## Data Availability

Not applicable.
